# Nosocomial malaria transmission risk in hospital wards in Abeokuta Nigeria: entomological and parasitological evidence from secondary healthcare facilities

**DOI:** 10.1186/s12936-026-05947-4

**Published:** 2026-05-27

**Authors:** Jumoke K. Fawole, Nofisat F. Lawal, Ayodele S. Babalola, David A. Ojo, Sammy O. Sam-Wobo, Olufunmilayo A. Idowu

**Affiliations:** 1 Department of Pure and Applied Zoology, Federal University of Agriculture Abeokuta, Alabata, Ogun State Nigeria; 2 Department of Microbiology, Federal University of Agriculture Abeokuta, Alabata, Ogun State Nigeria; 3 Department of Public Health and Epidemiology, Nigerian Institute of Medical Research, Yaba, Lagos, Nigeria

**Keywords:** Malaria, *Anopheles* mosquitoes, Sporozoites rate, Hospital-acquired infection, Malaria transmission, Africa

## Abstract

**Background:**

Malaria remains a major public health challenge in Nigeria, yet the potential for malaria transmission within healthcare facilities is poorly understood. This study investigated malaria prevalence among patients and caregivers, the presence of malaria vectors within hospital wards, and the potential for nosocomial malaria transmission in two secondary hospitals in Abeokuta, Ogun State.

**Methods:**

A cross-sectional study was conducted among inpatients admitted for conditions other than malaria across the Surgical/Orthopaedic, Pediatrics, and Obstetrics/Gynaecology wards. Finger-prick blood samples were collected from inpatients and caregivers who had stayed in the wards for at least three nights. Thick and thin blood smears were examined for Plasmodium falciparum. Information on participant demographics, duration of admission, and use of bed nets was also obtained. Indoor-resting mosquitoes were collected using mouth aspirators, identified morphologically, and examined for abdominal status, parity, blood-meal source, and P. falciparum sporozoites using ELISA.

**Results:**

A total of 134 participants were enrolled (80.6% patients; 19.4% caregivers). Malaria prevalence was high (74.6%), including 75.0% of patients and 73.1% of caregivers. Prevalence did not differ significantly by sex, ward type, or duration of admission (p > 0.05), and most infections involved low-density parasitaemia. Only 3.7% of participants reported using mosquito nets while on admission. A total of 714 mosquitoes were collected, including 80 Anopheles females. All Anopheles mosquitoes collected were members of the Anopheles gambiae s.l. Most Anopheles mosquitoes were fed (81.3%), all with human blood meals, and 38.8% were parous. Two Anopheles mosquitoes (2.5%) from the Obstetrics/Gynaecology ward tested positive for P. falciparum sporozoites.

**Conclusion:**

The coexistence of asymptomatic parasitaemic patients, infective mosquitoes, and inadequate protective measures indicates a plausible risk of nosocomial malaria transmission within hospital wards. Strengthening malaria-specific infection-prevention and vector-control strategies, including routine malaria screening, improved ward screening and treatment, environmental management, and universal access to insecticide-treated nets, is essential to protect vulnerable patients and support malaria control efforts in Nigeria.

## Background

Nosocomial infections remain a major concern in healthcare settings. According to [1], hospitalized patients who are infected or act as carriers of pathogenic microorganisms constitute potential sources of infection to healthcare workers, visitors, and other patients. Similarly, patients who acquire infections during hospitalization may further contribute to in-hospital transmission [2–4]. Exposure to pathogens within hospitals can occur through healthcare personnel, caregivers, contaminated environments, or other infected patients [5, 6]. While nosocomial infections are typically associated with bacterial and viral pathogens, mosquito-borne diseases such as malaria may also represent an under-recognized risk within hospital environments.

Malaria remains a critical public health emergency in Nigeria, which in 2024 contributed 24.3% of all global malaria cases and 30.3% of all global malaria deaths, the highest national burden of any country worldwide [7]. Nigeria also accounted for 38.6% of all global malaria deaths among children under five years of age, underscoring the disproportionate toll of the disease on the most vulnerable populations [7]. Malaria transmission is driven by the interaction between human reservoirs, competent *Anopheles* vectors, and environmental conditions that support mosquito survival and breeding [8, 9]. Hospitals are not exempt from these dynamics. Patients admitted with malaria or asymptomatic infections may serve as parasite reservoirs, while the presence of mosquito vectors within hospital environments creates opportunities for ongoing transmission.

Malaria is transmitted through the bite of infected female *Anopheles* mosquitoes. Of over 3,500 mosquito species described globally, approximately 400 belong to the genus *Anopheles* [10], with about 30 recognized as malaria vectors [1, 11]. In Nigeria, primary vectors belong to the *Anopheles gambiae* complex, while secondary vectors such as An. funestus, An. coustani, An. marshalli, and An. maculipalpis contribute to transmission, particularly during the dry season [9, 12]. Biting typically occurs at night, with species exhibiting either indoor or outdoor resting behaviour [13]. Mosquitoes become infectious after feeding on individuals carrying Plasmodium parasites—primarily P. falciparum, the dominant and most severe species in Nigeria [14].

In malaria-endemic regions such as Nigeria, transmission is sustained year-round, with peaks during the rainy season. Factors such as poor sanitation, overcrowding, poverty, and inadequate vector control contribute to persistent transmission [15]. These conditions may also extend into hospital environments, where water storage containers, drainage systems, poorly screened windows, and high human traffic can facilitate mosquito presence. Hospitalized individuals with malaria, particularly in paediatric and general wards, may therefore act as continuous sources of infection for mosquitoes entering these environments [5].

Although malaria is not traditionally classified as a nosocomial infection, increasing evidence suggests that in-hospital transmission is possible, especially in high-burden settings where vectors are present [5, 16, 17]. In such contexts, transmission may occur through both iatrogenic routes (e.g., needlestick injury or transfusion) and vector-mediated pathways, where infected patients serve as reservoirs and *Anopheles* mosquitoes gain access to hospital wards. However, data on vector abundance, species composition, and infective status within hospital environments in Nigeria remain limited.

Ogun State, where this study was conducted, lies within a meso- to hyper-endemic malaria transmission zone. Malaria prevalence in the state increased from 14.7% in 2015 to 24.9% in 2021 [18], indicating rising transmission intensity. Additional indicators, such as spleen rates, further reflect sustained endemicity, with reported values of 15.5% in Edo South [19] and 12.9% in southwestern Nigeria, where splenomegaly was significantly associated with malaria parasitaemia [20]. These findings highlight the persistence of asymptomatic parasite reservoirs within the population, including among hospitalized individuals.

Therefore, this study aimed to determine the prevalence of malaria infection among patients hospitalized for non-malaria conditions, assess mosquito vector abundance within hospital wards in Abeokuta, Ogun State, and evaluate the presence of infective Plasmodium sporozoites in these mosquitoes. The ensuing findings provide important insights into the potential for undetected malaria transmission within healthcare facilities.

## Methods

### Study area

**Fig. 1:**
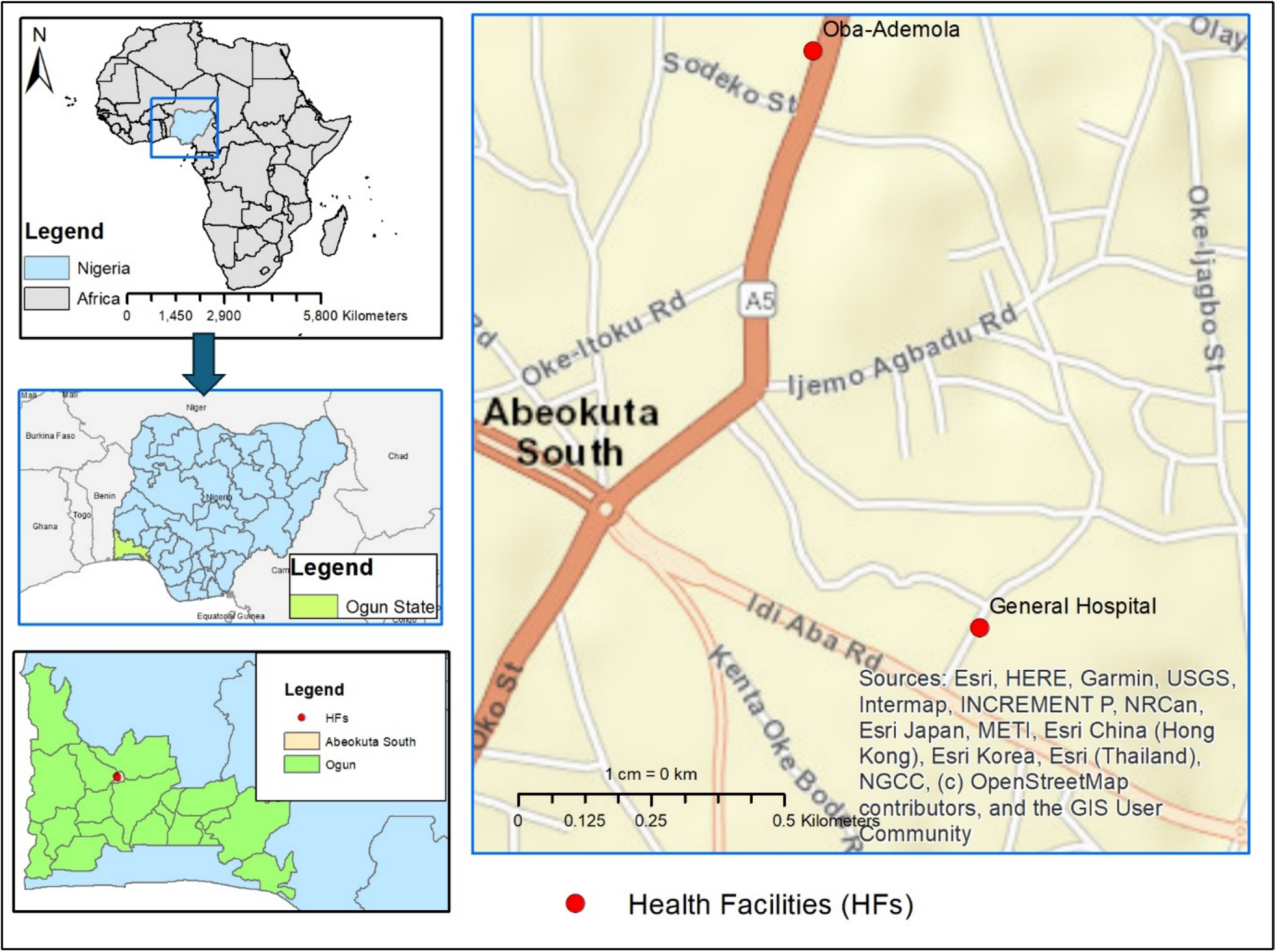
Map showing the study areas in Africa, Nigeria and Ogun State

This study was conducted in Abeokuta, the capital of Ogun State, Nigeria, between May and July 2018. Abeokuta is located at approximately longitude 7°15′N and latitude 3°21′E. It is situated on the east bank of the Ogun River near a group of rocky outcrops in a wooded savannah, 77 km north of Lagos by railway [6]. Two hospitals were used for this study: Oba Ademola Maternity Hospital and State Hospital, Sokenu–Ijaiye, both situated in the Abeokuta South Local Government Area of Ogun State (Fig. 1). Malaria vectors are resistant to WHO diagnostic concentrations of insecticides in the study area [8, 21]

### Inclusion and exclusion criteria

Blood samples were collected from inpatients and caregivers who had stayed in the hospital wards for at least three consecutive nights. Patients admitted primarily for malaria treatment, outpatients, and caregivers who did not stay overnight in the wards were excluded from the study.

### Sampling method

A combination of purposive and convenience sampling was used. All individuals present in the wards during the study period were approached, and those who met the eligibility criteria and provided informed consent were enrolled. A total of 134 participants (108 inpatients and 26 caregivers) were included.

Eligibility was restricted to individuals who had stayed in the wards for at least three consecutive nights to ensure adequate exposure to the hospital environment. Patients admitted primarily for malaria, outpatients, and non-resident caregivers were excluded. The final sample represents all eligible and consenting ward occupants during the study period, consistent with non-probability sampling approaches commonly used in hospital-based studies [22, 23]. All operational inpatient wards accessible during the study period were included. At the maternity hospital, the obstetrics ward was the only inpatient unit, while at the second facility, obstetrics ward, paediatric and surgical/orthopaedic wards were the main accessible inpatient wards.

### Blood sample collection and microscopic examination

Blood samples were collected via finger prick using sterile lancets to prepare thick and thin blood smears for malaria parasite detection [24]. Slides were stained with 10% Giemsa for 15 min and examined under × 100 oil immersion microscopy. A thick smear was considered negative if no parasites were observed after examining a minimum of 100 high-power fields.

Parasite density was estimated using the parasite-to-white blood cell (WBC) method, with counts expressed as parasites per microlitre of blood assuming a standard WBC count [25]. For quality control, all slides were independently re-examined by a second experienced microscopist who was blinded to participant information and the results of the primary reader. Where the primary and secondary readers agreed, the result was recorded as final. In cases of discordance (defined as disagreement on parasite presence or absence, species identification, or parasite density) slides were referred to a third independent expert microscopist serving as a tiebreaker, who was similarly blinded to participant details and prior read results. Results in discordant cases were determined by the majority read across the three microscopists.

Basic demographic and clinical data, including age, sex, cause of admission, and duration of hospital stay, were recorded prior to sample collection. Participants testing positive were referred for clinical management according to national malaria treatment guidelines [26].

### Mosquito collection and entomological procedures

Mosquito collections were conducted between May and July 2018 across hospital wards using an aspirator, selected for its suitability for indoor resting mosquito sampling in clinical environments [27]. Collections were carried out twice weekly between 04:00 and 06:00 h, in accordance with ethical considerations governing the use of the hospital for research. All mosquitoes were labelled by date, ward, and time of collection, and transported to the laboratory for identification, gonotrophic and parity assessment, blood-meal analysis, and sporozoite detection following standard WHO entomological guidelines. Mosquitoes were identified morphologically to the genus level using standard taxonomic keys,molecular identification of sibling species within the *Anopheles* gambiae complex was not performed [28, 29].

### Gonotrophic and parity status assessment

Gonotrophic status was determined based on abdominal appearance and classified as unfed, fed, semi-gravid, or fully gravid. Parity was assessed by ovarian dissection using the Detinova [30] technique. Mosquitoes were dissected under a microscope, and tracheolar coiling patterns were used to classify individuals as nulliparous or parous. Assessments were made only where tracheolar structures were clearly visible, including in semi-gravid and gravid specimens where possible.

### Blood meal analysis and sporozoite detection

Blood meal sources were determined using the direct precipitin test as described by Collins et al. [31]. Gut contents were tested with anti-human globulin, and visible precipitation indicated human blood. Detection of *Plasmodium falciparum* sporozoites was performed using ELISA on mosquito head–thorax homogenates following established protocols [32, 33]. Samples exceeding the optical density threshold were classified as positive.

### Data analysis

Data generated were entered into excel and further analyzed using SPSS version 23 (IBM Corps). Differences in prevalence across different in-patient wards, gender and length of admission were analyzed using chi-square. Results were presented using tables and figures, using either excel or ggplot function in R programming software (Version 4.3). p-values < 0.05 were considered to be significant.

## Results

### Summary of participant characteristics

A total of 134 participants were enrolled across the three wards, including 108 inpatients (80.6%) and 26 caregivers (19.4%). Females predominated (87.3%), largely reflecting the Obstetrics/Gynaecology ward, where all participants were female. Most participants (71.6%) had been admitted for ≤ 7 days, although longer stays were more common in the Surgical/Orthopaedic ward (Table 1).

**Table 1:** Characteristics of study population based on sex, duration of hospital admission and malaria prevalence in relation to different wards and categories of participants

Ward	Participants N (%)	No Examined N (%)	Gender N (%)		Duration of admission N (%)		+ ve for Malaria N (%) (95% CI)
			Male	Female	≤ 7 days	** > 7 **days	
Surgical/ Orthopaedic *p* = *0.980*	Patients	26(78.8)	10(38.5)	16(61.5)	5(19.2)	21(80.8)	22(84.6) (70.7–98.0)
	Caregivers	07(21.2)	0(0.0)	7(100.0)	6(85.7)	1(14.3)	6(85.7) (59.8–100.0)
	Total	33(24.6)	10(30.3)	23(69.7)	11(33.3)	22(66.7)	28(84.8) (72.6–97.0)
Paediatrics *p* = *0.613*	Patients	14(63.6)	6(42.9)	8(57.1)	9(64.3)	5(35.7)	10(71.4) (47.8–95.0)
	Caregivers	08(36.4)	1(12.5)	7(87.5)	8(100.0)	0(0.0)	7(87.5) (64.6–100.0)
	Total	22(16.4)	7(31.8)	15(68.2)	17(77.3)	5(22.7)	17(77.3) (59.8–94.8)
Obstetric/ Gyneacology *p* = *0.295*	Patients	68(86.1)	0(0.0)	68(100.0)	58(85.3)	10(14.7)	49(72.1) (61.5–82.7)
	Caregivers	11(13.9)	0(0.0)	11(100.0)	10(90.9)	1(9.1)	6(54.5) (25.1–83.9)
	Total	79(59.0)	0(0.0)	79(100.0)	68(86.1)	11(13.9)	55(69.6) (59.5–79.7)
Overall p = *0.807*	Patients	108(80.6)	16(14.8)	92(85.2)	72(66.7)	36(33.4)	81(75.0) (66.8–83.2)
	Caregivers	26(19.4)	1(3.8)	25(96.2)	24(92.3)	2(7.7)	19(73.1) (56.1–90.1)
	Total	134(100.0)	17(12.7)	117(87.3)	96(71.6)	38(28.4)	100(74.6) (67.2–82.0)

Overall, malaria prevalence was high (74.6%), with similar positivity among inpatients (75.0%) and caregivers (73.1%). Prevalence was highest in the Surgical/Orthopaedic ward (84.8%), followed by Paediatrics (77.3%) and Obstetrics/Gynaecology (69.6%). However, these differences were not statistically significant, either across wards or between participant groups (p > 0.05). The relatively small number of caregivers compared to inpatients may have limited the statistical power to detect differences between these groups.

Notably, only 3.7% of patients reported sleeping under a mosquito bed net during admission.

### Malaria prevalence by participant characteristics

**Fig. 2:**
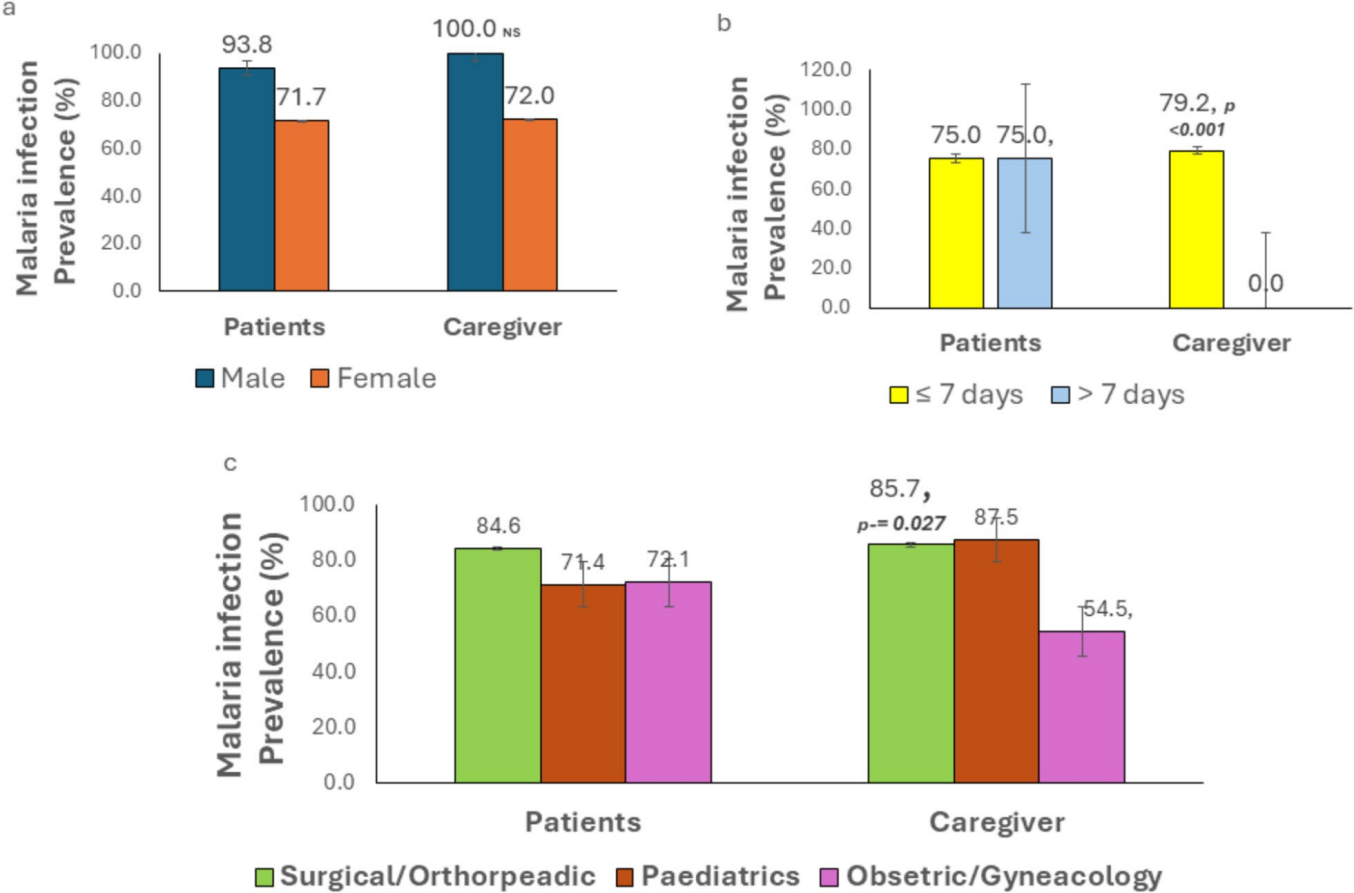
Malaria prevalence by participant characteristics **a** gender **b** length of admission (days) **c** ward type. NS means no significant difference (p > 0.05) with respect to length of admission in days

Malaria prevalence was consistently high across all participant groups (Fig. 2). There were no significant differences by sex or among patients based on duration of admission (p > 0.05). However, among caregivers, malaria prevalence was significantly higher in those with ≤ 7 days of stay compared with > 7 days (p < 0.0001). Prevalence also differed significantly across wards among caregivers (p = 0.027), while remaining comparable across wards for inpatients.

Gametocyte carriage was detected in 12 of the 134 participants (approximately 9.0%), representing the subpopulation with the potential to infect host-seeking *Anopheles* mosquitoes within the ward environment. Of the 12 gametocyte-positive individuals, 10 were from the Obstetrics/Gynaecology ward and 2 were from the Paediatric ward. The concentration of gametocyte carriers in the Obstetrics/Gynaecology ward suggests that this ward may represent a particularly high-risk environment for mosquito-to-human and human-to-mosquito transmission within the hospital setting.

### Overall malaria prevalence by length of admission across hospital wards

**Fig. 3:**
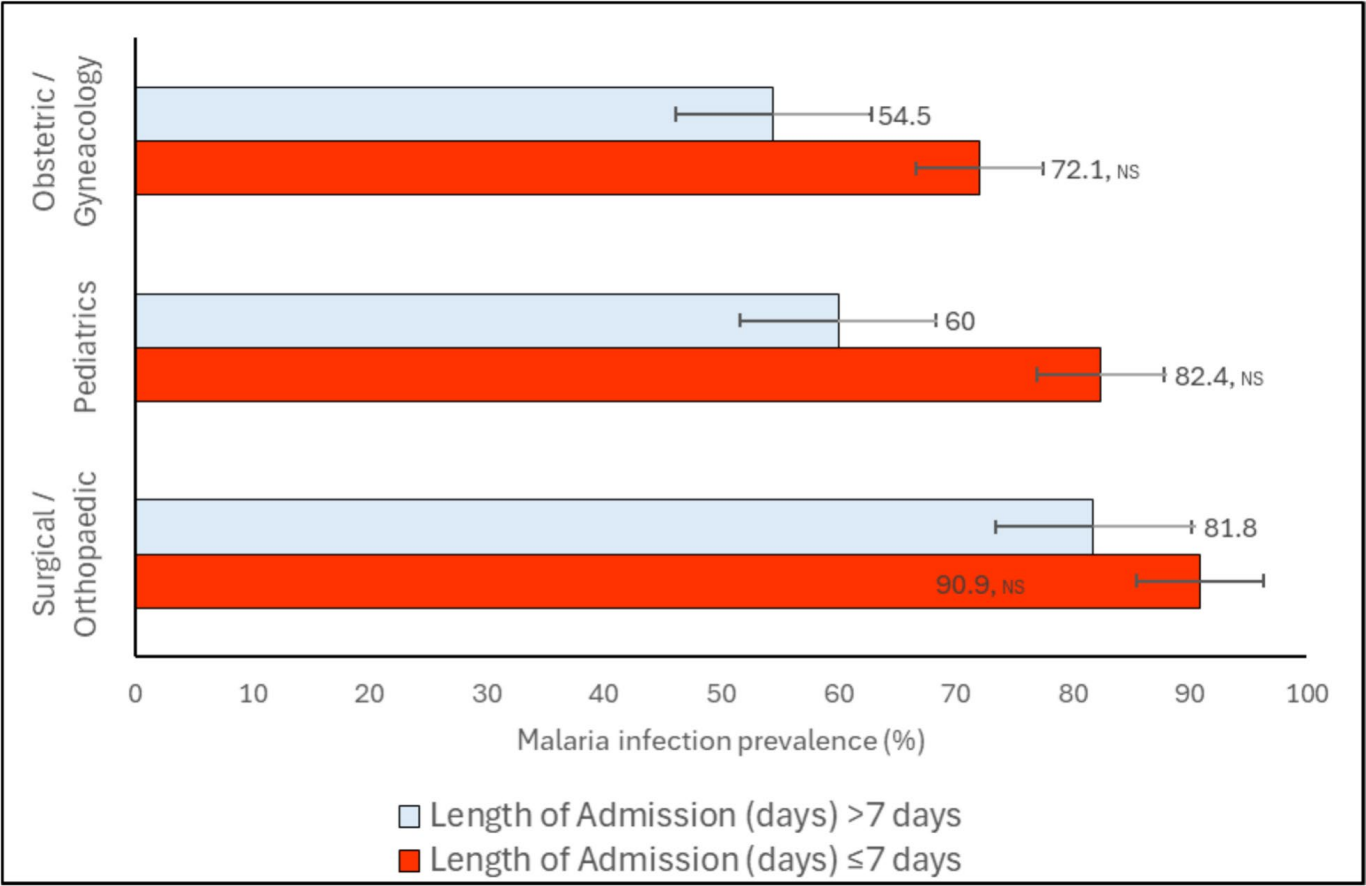
Malaria prevalence by length of admission across hospital wards. NS means no significant difference (p > 0.05) with respect to length of admission in days

Malaria prevalence was high across all wards regardless of duration of admission (Fig. 3). Although participants admitted for ≤ 7 days showed slightly higher positivity across wards, these differences were not statistically significant (p > 0.05), indicating no association between length of hospital stay and malaria infection.

### Distribution of parasitaemia levels among malaria-positive participants by hospital ward and duration of admission

**Fig. 4:**
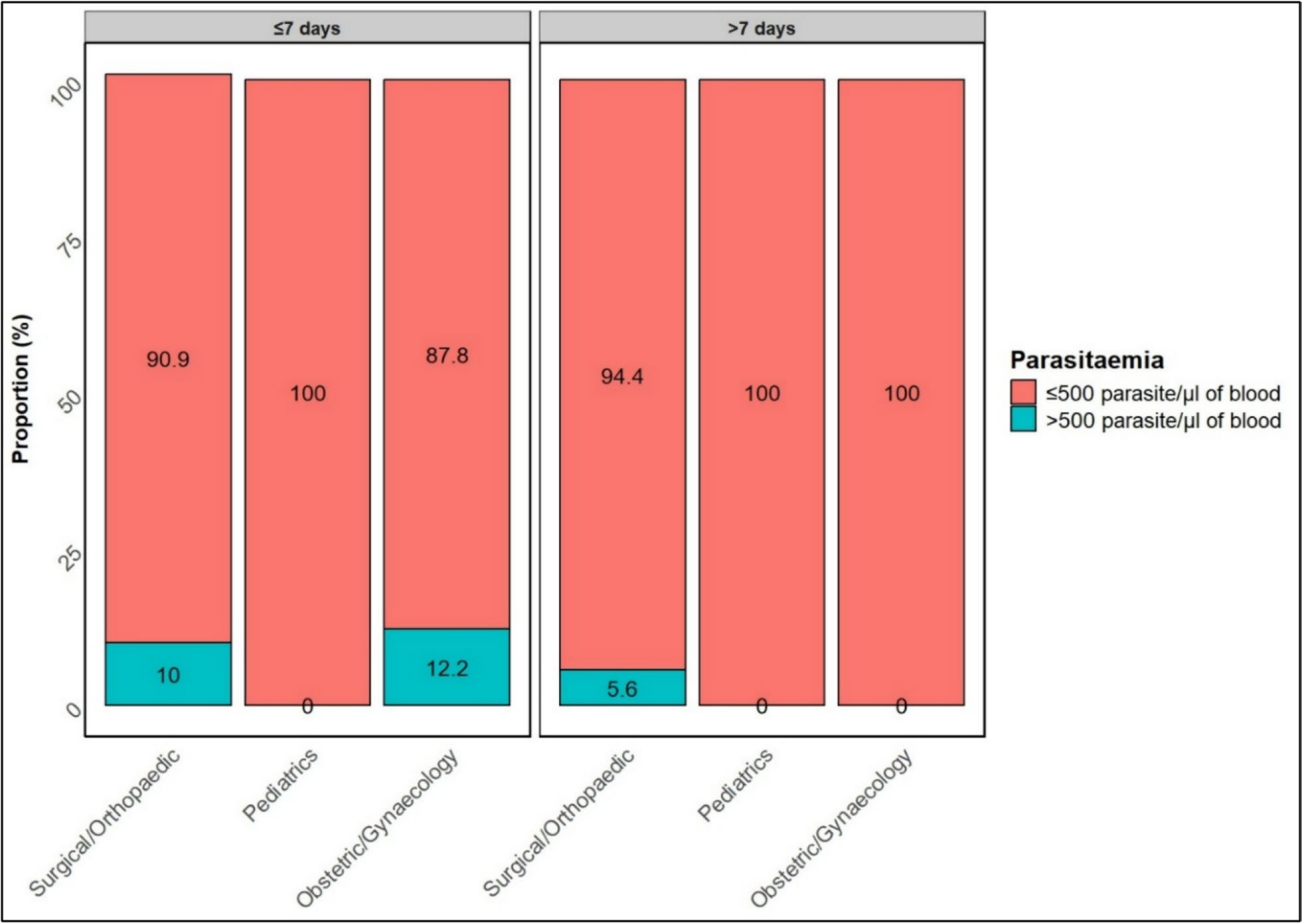
Distribution of parasitaemia levels among malaria-positive participants by hospital ward and duration of admission

Across all participant groups, low-density parasitaemia predominated, with relatively few cases of moderate or high parasitaemia (Fig. 4). This pattern was consistent across wards and duration-of-stay categories.

### Prevalence of mosquito vectors in the study area

A total of 714 mosquitoes were collected, of which 80 (11.2%) were *Anopheles gambiae* s.l. (Table 2). Mosquitoes were distributed across all wards, with no significant differences in abundance between wards (p = 0.059). (Table 2).

**Table 2:** Occurence of mosquitoes in the study areas

Ward	Anophelines N (%)	95% CI	Culicines N (%)	95% CI	Total
Surgical/Orthopaedic	15 (9.3)	4.8–13.8	146 (90.7)	86.2–95.2	161
Children	6 (7.1)	1.6–12.6	78 (92.9)	87.4–98.4	84
Obstetrics/Gynaecology	59 (12.6)	9.6–15.6	410 (87.4)	84.4–90.4	469
Total	80 (11.2)	8.9–13.5	634 (88.8)	86.5–91.1	714

All the Anophelines collected were members of the *Anopheles gambiae s.l*

### Abdominal condition and parity classification of *Anopheles* Mosquitoes Collected from different wards

Most *Anopheles* mosquitoes were blood-fed (81.3%), and all blood meals were of human origin. Smaller proportions were semi-gravid (13.8%) or fully gravid (5.0%) (Table 3). Parity analysis showed that 38.8% were parous and 61.2% nulliparous, indicating a relatively young mosquito population, with similar patterns observed across wards (Table 3).

**Table 3:** Abdominal Condition, parity and infection status of *Anopheles* Mosquitoes Collected from Different Wards

Wards	*Anopheles*	Abdominal Condition			Parity		Number infected
		Fed	Half gravid	Fully gravid	Parous	Nulliparous	
Surgical/ Orthopaedic	15	13 (86.7)	2 (13.3)	0 (0.0)	7 (46.7)	8 (53.3)	0 (0.0)
Children	06	5 (83.3)	1 (16.7)	0 (0.0)	2 (33.3)	4 (66.7)	0 (0.0)
Obstetrics/ Gynecology	59	47 (79.7)	8 (13.5)	4 (6.8)	22 (37.3)	37 (62.7)	2 (3.4)
Total	80	65 (81.3)	11 (13.7)	4 (5.0)	31 (38.8)	49 (61.2)	2 (2.5)

All the Anophelines collected were members of the *Anopheles gambiae s.l*

### Plasmodium falciparum sporozoite rate in *Anopheles* mosquitoes

The overall prevalence of *Plasmodium* sporozoites among the *Anopheles* mosquitoes collected across the hospital was 2.5%, with all positive mosquitoes detected in the Obstetrics/Gynaecology ward. No sporozoite-positive mosquitoes were found in the Surgical/Orthopaedic or Pediatrics wards at the time of collection (Table 3).

## Discussion

This study demonstrates a high Plasmodium falciparum prevalence among both inpatients and caregivers in secondary hospitals in Abeokuta, Nigeria. The overall prevalence (74.6%) exceeds that reported in Ibadan [34] but is slightly lower than the 80.9% reported by Simon-Oke et al. [35]. These findings indicate sustained malaria transmission in the region despite ongoing control efforts. The high burden of asymptomatic parasitaemia is consistent with patterns observed in southwestern Nigeria and reflects persistent transmission intensity. Although Nigeria’s NMEP implements LLINs, IRS, SMC, and IPTp at scale [7], hospital environments are not routinely included in vector control strategies. These findings highlight the relevance of extending surveillance and preventive measures to inpatient settings, where parasitaemic individuals and mosquito vectors may coexist.

The consistently high prevalence across sex, ward type, and participant groups suggests that most infections were likely community-acquired prior to admission. The predominance of low-density parasitaemia aligns with partial immunity typical of high-transmission settings [36, 37, 38, 39, 40]., Although infections were asymptomatic at the time of sampling, higher parasite densities may still pose a risk of progression to clinical disease, particularly among vulnerable inpatients [38, 48]. Longitudinal studies are required to better define the clinical implications of asymptomatic infections in hospital populations.

The higher malaria prevalence observed among short-stay caregivers likely reflects undetected community-acquired infections at admission rather than in-hospital acquisition. However, the detection of sporozoite-positive *Anopheles* gambiae s.l. within wards indicates that exposure to infective vectors can occur within the hospital environment. Even brief exposure may result in infection, highlighting the importance of routine malaria screening for both patients and caregivers at admission. Caregivers, who are often not included in hospital infection prevention protocols, may act as bridge populations linking community and facility transmission.

The presence of gametocyte carriers (9.0%) further supports the potential for transmission within hospital environments. As the transmissible stage of the parasite, gametocytes enable human-to-mosquito infection, and their coexistence with infective vectors suggests that conditions for bidirectional transmission may be present [41]. The higher concentration of gametocyte carriers in the Obstetrics/Gynaecology ward may indicate localized transmission risk and warrants further investigation.

Entomological findings reinforce the possibility of vector–host contact within hospital wards. *Anopheles* mosquitoes were collected from all wards, and all blood meals were of human origin, confirming human–vector interaction in these settings. While this may reflect environmental or structural factors that facilitate mosquito entry and persistence, these were not directly assessed in this study. Similar patterns have been reported in other West African settings [42, 43].

The presence of parous mosquitoes (38.8%) and sporozoite-positive individuals (2.5%) indicates that vectors capable of sustaining transmission were present within wards. Although the sporozoite rate was low, even small numbers of infective mosquitoes may contribute to transmission risk [44, 45]. The detection of infective vectors in the Obstetrics/Gynaecology ward is of particular concern given the increased vulnerability of pregnant women and neonates to malaria-related complications [6, 38, 48]. This pattern may reflect host-related or environmental factors, including increased attractiveness of pregnant women to *Anopheles* mosquitoes [46, 47] or ward-specific conditions that favour mosquito entry. However, these explanations remain speculative and require further study.

From a health systems perspective, these findings highlight a potential gap in malaria control. Notably, only 3.7% of patients reported using insecticide-treated nets during admission, indicating limited use of personal protective measures within hospital wards. In the presence of parasitaemic individuals and infective vectors, this low level of protection suggests conditions that may permit transmission within healthcare settings. While national malaria control strategies primarily target community transmission, these findings support consideration of facility-based interventions, including routine screening at admission, provision of insecticide-treated nets for inpatient use, improved ward screening, and enhanced environmental management.

## Study limitations

This study has several limitations. First, it was conducted in only two hospitals within a single urban area, which may limit the generalizability of the findings. Second, as participants were not tested at admission, it was not possible to distinguish community-acquired infections from potential in-hospital transmission. Third, the relatively small number of caregivers compared to inpatients may have limited the statistical power to detect differences between these groups.

Mosquito collections relied on aspiration and may not fully capture vector density or species diversity across ward microenvironments. In addition, environmental factors such as nearby breeding sites were not systematically assessed. Future studies should incorporate larval habitat characterization around hospital facilities, including larval sampling and species identification, to support targeted larval source management and complement indoor surveillance.

Although parity was determined for all specimens, assessments in semi-gravid and gravid females may be less precise due to partial obscuration of tracheolar structures, potentially introducing minor classification uncertainty. Furthermore, species identification was based on morphological methods, and molecular differentiation of sibling species within the *Anopheles* gambiae complex was not performed, limiting assessment of species-specific transmission dynamics.

Despite these limitations, the study provides important insights into the overlooked risk of malaria transmission within hospital settings and highlights critical gaps in facility-level malaria control.

## Conclusion

This study demonstrates a high burden of malaria infection among hospital inpatients and caregivers in Abeokuta, alongside the presence of infective *Anopheles* mosquitoes within hospital wards. The coexistence of asymptomatic parasitaemic individuals, active mosquito vectors, and suboptimal protective measures suggests a potential risk of hospital-based malaria transmission, although the absence of baseline screening at admission precludes definitive distinction between community- and hospital-acquired infections. While most infections were likely community-acquired, the detection of sporozoite-positive mosquitoes and gametocyte carriers indicates that health facilities may contribute to sustaining transmission risk, particularly among vulnerable groups such as pregnant women and neonates.

These findings underscore the need for strengthened facility-based malaria prevention strategies. In addition to routine screening at admission, hospitals should implement structural and operational vector control measures, including improved window and ward screening, provision of insecticide-treated nets for inpatient use, and enhanced environmental management to reduce mosquito entry and breeding. Embedding these interventions within hospital infection prevention policies could substantially reduce transmission risk and support broader malaria control and elimination efforts in Nigeria.

## Data Availability

All data generated and analyzed during the study are present in the article.

## Abbreviations

WHO: World Health Organization
IPC: Infection prevention and control
ELISA: Enzymes linked immunosorbent assay
ITNs: Insecticide treated nets
ECDCP: European centre for disease control and prevention
CDC: Centre for diseases control
CSP: Circumsporozoite protein
PacMOSSI: Pacific mosquito surveillance, strengthening impact
